# Supplementary material for: Short-term outcomes of cochlear implantation for single-sided deafness compared to bone conduction devices and contralateral routing of sound hearing aids—Results of a Randomised controlled trial (CINGLE-trial)
Source: PLoS One. 2021 Oct 13;16(10):e0257447. doi: 10.1371/journal.pone.0257447 (PMC8513831; doi:10.1371/journal.pone.0257447)
Supplement: S1 File — (PDF) [file pone.0257447.s001.pdf]

RESEARCH PROTOCOL

NL45288.041.13

CINGLE-TRIAL:

COCHLEAR IMPLANTATION FOR SINGLE-SIDED DEAFNESS

(VERSION 3, APRIL 2ND, 2014)

UMC Utrecht

PROTOCOL TITLE: *'CINGLE-TRIAL: COCHLEAR IMPLANTATION FOR SINGLE-SIDED DEAFNESS'*

**Protocol ID**	**NL45288.041.13**
**Short title**	'CINGLE-TRIAL: COCHLEAR IMPLANTATION FOR SINGLE-SIDED DEAFNESS'
**Version**	3
**Date**	April 2nd, 2014
**Coordinating investigator/project leader**	Prof. dr. W. Grolman<br>Chair of department of Otorhinolaryngology<br>University Medical Center Utrecht<br>Ph. +31 88 75 566 44<br>W.Grolman@umcutrecht.nl
**Principal investigator(s) (in Dutch: hoofdonderzoeker/ uitvoerder)**	**Principal investigators/local executor:**<br>UMC Utrecht (only site):<br>Prof. dr. W. Grolman<br>Chair of department of Otorhinolaryngology<br>University Medical Center Utrecht<br>Ph. +31 88 75 566 44<br>W.Grolman@umcutrecht.nl<br><br>**Researchers:**<br>Drs. H.P.M. Peters, researcher<br>Department of Otorhinolaryngology<br>University Medical Center Utrecht<br>Ph. +31 88 75 56644<br>h.p.m.peters-3@umcutrecht.nl<br><br>Drs. A. van Zon, researcher<br>Department of Otorhinolaryngology<br>University Medical Center Utrecht<br>Ph. +31 88 75 56644<br>a.vanzon@umcutrecht.nl

	Drs. A.L. Smit, otorhinolaryngologist<br>Department of Otorhinolaryngology<br>University Medical Center Utrecht<br>Ph. +31 88 75 566 44<br>a.l.smit-9@umcutrecht.nl<br><br>Dr. I. Stegeman, epidemiologist<br>Department of Otorhinolaryngology<br>University Medical Center Utrecht<br>Ph. +31 88 75 58375<br>i.stegeman@umcutrecht.nl<br><br>Dr. G.A. van Zanten, audiologist<br>Department of Otorhinolaryngology<br>University Medical Center Utrecht<br>Ph. +31 88 75 57574<br>g.a.vanzanten@umcutrecht.nl
**Sponsor (in Dutch: verrichter/opdrachtgever)**	University Medical Center Utrecht<br>Department of Otorhinolaryngology<br>Ph. +31 88 75 566 44<br>W.Grolman@umcutrecht.nl
**Independent physician(s)**	Drs. I. Ligtenberg-Van der Drift, otorhinolaryngologist<br>Department of Otorhinolaryngology<br>University Medical Center Utrecht<br>Ph. +31 88 75 566 44<br>I.Ligtenberg-vanderDrift@umcutrecht.nl
**Laboratory sites <*if applicable*>**	n/a
**Pharmacy <*if applicable*>**	n/a

PROTOCOL SIGNATURE SHEET

Name	Signature	Date
**For non-commercial research,**<br>**Head of Department:**<br><br>Prof. dr. W. Grolman<br>Chair of department of Otorhinolaryngology,<br>University Medical Center Utrecht	[signature]	2/4/14
**Coordinating Investigator/Project leader/Principal Investigator:**<br><br>Prof. dr. W. Grolman<br>Chair of department of Otorhinolaryngology,<br>University Medical Center Utrecht	[signature]	2/4/14

TABLE OF CONTENTS

LIST OF ABBREVIATIONS AND RELEVANT DEFINITIONS 7
SUMMARY 8

1. INTRODUCTION AND RATIONALE 10
  1.1 Introduction 10
  1.2 Rationale 11
2. OBJECTIVES 14
3. STUDY DESIGN 15
4. STUDY POPULATION 17
  4.1 Population (base) 17
  4.2 Inclusion criteria 17
  4.3 Exclusion criteria 17
  4.4 Sample size calculation 17
5. TREATMENT OF SUBJECTS 17
  5.1 Investigational product/treatment 17
  5.2 Use of co-intervention 17
6. METHODS 17
  6.1 Study parameters/endpoints 17
    6.1.1 Main study parameter/endpoint 17
    6.1.2 Secondary study parameters/endpoints 17
  6.2 Randomisation, blinding and treatment allocation 17
  6.3 Study procedures 17
    6.3.1 Objective 17
    6.3.2 Subjective 17
  6.4 Withdrawal of individual subjects 17
    6.4.1 Specific criteria for withdrawal 17
  6.5 Replacement for individual subjects after withdrawal 17
  6.6 Follow up of subjects withdrawn from treatment 17
  6.7 Premature termination of the study 17
  6.8 End of study 17
7. SAFETY REPORTING 17
  7.1 Section 10 WMO event 17
  7.2 Adverse and serious adverse events 17
  7.3 Follow up of serious adverse events 17
  7.4 Monitoring 17
8. STATISTICAL ANALYSIS 17
9. ETHICAL CONSIDERATIONS 17
  9.1 Regulation Statement 17
  9.2 Recruitment and consent 17
  9.3 Benefits and risks assessment, group relatedness 17
  9.4 Compensation for injury 17

9.5 Incentives 17
10. ADMINISTRATIVE ASPECTS AND PUBLICATION 17
10.1 Handling and storage of data and documents 17
10.2 Amendments 17
10.3 Annual progress report 17
10.4 End of study report 17
10.5 Public disclosure and publication policy 17

11. REFERENCES 17

LIST OF ABBREVIATIONS AND RELEVANT DEFINITIONS

APHAB	Abbreviated Profile of Hearing Aid Benefit questionnaire
AE	Adverse event
BAHA	Bone-Anchored Hearing Aid
CCMO	Central Committee on Research Involving Human Subjects
CI	Cochlear Implant
CVC	Consonant-vowel-consonant
CvZ	College voor Zorgverzekeraars (Dutch: association of health care insurance companies)
CROSS	Contralateral Routing of Sound System
ENT	Ear Nose Throat
EQ5D	Euro-QoL 5D questionnaire
GBI	Glasgow Benefit Inventory
HUI(3)	Health Utilities Index (version 3) questionnaire
IC	Informed Consent
METC	Medical research ethics committee (MREC) (in Dutch: *Medisch Ethische Toetsingscommissie* (METC))
QALY	Quality Adjusted Life Year
QoL	Quality-of-Life
RCT	Randomised Controlled Trial
SAE	Serious adverse event
SNR	Signal-to-noise-ratio
SSD	Single-Sided Deafness
SSQ	Speech, Spatial and Quality of Hearing Scale
SUSAR	Suspected Unexpected Serious Adverse Reaction
THI	Tinnitus Handicap Inventory
TQ	Tinnitus Questionnaire
TTO	Time Trade Off
VAS	Visual Analogue Scale
UMC (Utrecht)	University Medical Center (Utrecht)
Wbp	Personal Data Protection Act (in Dutch: *Wet Bescherming Persoonsgegevens*)
WMO	Medical Research Involving Human Subjects Act (in Dutch: *Wet Medisch-wetenschappelijk Onderzoek met mensen*)

SUMMARY

Rationale: Patients who develop single-sided deafness (SSD) become aware of the importance of hearing with two ears in everyday listening environments. Current clinical practice for patients with SSD consists of optimizing hearing using a Bone-Anchored Hearing Aid (BAHA) or a Contralateral Routing of Sound System (CROSS). With both devices sound awareness on the deaf side can be improved, but they do not provide bilateral auditory input, which is needed to achieve the actual benefits of hearing with two ears. These limitations may be overcome by providing a cochlear implant (CI) and consequently generating auditory input to the affected ear.

Objective: The objectives of this study are to evaluate the clinical outcomes gained from having a cochlear implant (CI) over standard health care therapy with either BAHA or CROSS in patients with SSD and to examine the cost utility of cochlear implantation in these patients.

Study design: 120 subjects with acute (≥3 months and ≤10 years onset) SSD will be included in this Randomised Controlled Trial (RCT) after their informed consent. 30 Subjects shall receive a CI on the deaf side after randomisation (Group A). The other 90 subjects shall start with a 6-week during test period with either a BAHA on a headband (n = 45, Group B) or with a CROSS (n = 45, Group C). After these 6 weeks, patients in group B switch to a test period with a CROSS for 6 weeks and vice versa for patients in group C. After completing both test periods patients in group B and group C will choose for further treatment with a CROSS, a definitive surgically implanted BAHA or no treatment. The follow-up sessions will take place 6, 12, 18, 24, 36, 48 and 60 months after randomisation for participants in all groups.

Study population: 120 subjects aged 18 or older with postlingual SSD who are eligible for cochlear implantation.

Intervention (if applicable): Cochlear Implantation versus BAHA or CROSS

Main study parameters/endpoints: The main outcome will be the performance on the modified Plomp hearing test. Secondary outcome measures will be: performance on the Standard Dutch phoneme test (NvA-list), the Speech intelligibility test with spatially separated sources, the Crescent of sound test (Quentin Summerfield), self-reported benefits in

everyday listening situations and quality of life assessed with the Speech, Spatial and Qualities Hearing Scale (SSQ), Abbreviated Profile of Hearing Aid Benefit (APHAB), Health Utilities Index (HUI3), Glasgow Benefit Inventory (GBI), Hospital Anxiety and Depression Score (HADS), Time Trade-Off (TTO), Visual Analogue Scales (VAS) and EuroQol-5D (EQ5D) and tinnitus questionnaires (Tinnitus Handicap Inventory [THI], Tinnitus Questionnaire [TQ] and Tinnitus Burden Questionnaire). Participants will keep a monthly diary to assess cost utility.

Nature and extent of the burden and risks associated with participation, benefit and group relatedness:
The study is considered a non-significant risk evaluation of routine modalities for SSD in clinical practice (BAHA and CROSS) compared to a modality, cochlear implantation, which is already commonly clinically applied in patients with bilateral hearing loss. The evaluation consists of 8 test sessions of 2 hours each, spread out over five years of time, excluding the monthly cost diary. Furthermore, subjects in group B and C will complete three additional questionnaires in their BAHA and CROSS test periods.

1. INTRODUCTION AND RATIONALE

1.1 Introduction

The present study was initiated by Prof. Dr. W. Grolman (University Medical Center Utrecht) who approached Niels van Druten (Cochlear® Benelux NV) in 2013 for a research collaboration on adult cochlear implantation in patients with single-sided deafness (SSD) with the idea of using a Randomised Controlled Trial (RCT) to compare cochlear implantation to current clinical practice with either a Bone-Anchored Hearing Aid (BAHA) or a Contralateral Routing of Sound System (CROSS).

Patients who develop SSD become aware of the importance of hearing with two ears in everyday listening environments. Current treatment modalities for SSD (i.e. BAHA or CROSS) are effective in restoring sound awareness to the deaf side, but they do not provide benefits with regard to sound localization and improvement in speech perception in noise. These limitations may be overcome by providing a cochlear implant (CI) to the affected ear. The main objective of our study is to evaluate the clinical outcome after cochlear implantation compared to standard health care therapy with either CROSS or BAHA in patients with SSD.

There is no high quality research on the topic of cochlear implantation for patients with single-sided deafness yet. Although current available evidence is limited, the first results of non-randomised trials are promising. In patients with SSD, cochlear implantation leads to an improved quality of life, better speech perception, localization of sounds and reduction of tinnitus complaints compared to BAHA, CROSS or no treatment (Arndt 2010; vd Heyning 2008; Távora-Vieira 2013). Conversely, the significant costs of cochlear implantation over CROSS or BAHA limit the potential use of this modality in patients with SSD. Further high quality research is certainly necessary to provide compelling evidence of sufficiently large benefits of cochlear implantation over CROSS or BAHA in patients with SSD to justify the additional costs. Therefore, our study will be conducted as a RCT to control for biases when assigning subjects to one of three study groups. RCTs are the most rigorous experimental design for establishing the effectiveness of one intervention compared with another (Woolf 1990).

The study is considered a non-significant risk evaluation of routine modalities for SSD in clinical practice (BAHA and CROSS) compared to cochlear implantation, which is already commonly clinically applied in patients with bilateral hearing loss. The risks associated with CROSS, BAHA and cochlear implantation are discussed in Chapter 7.2.

In the rest of the protocol "CROSS", "BAHA" and "CI" respectively will refer to the technical details and software settings as follows:

- CROSS: (BI)CROSS (clinical practice)
- BAHA: Cochlear® BAHA system 4
- CI: Cochlear® processor system 6 with the *Custom Sound* software

This research will be conducted according to the study details outlined in this protocol and in accordance with Good Clinical Practice (GCP) and finally Dutch legislation.

1.2 Rationale

SSD, or unilateral sensorineural hearing loss, refers to significant or total hearing loss in one ear. Patients who develop SSD become aware of the importance of binaural hearing in everyday listening environments. They experience difficulty in following conversations on their deaf side, in understanding speech in background noise and in determining the localization of sounds (Giolas 1994). Patients with SSD experience a significant disability in auditory function that affected their speech perception, communication and social interaction (Wie 2010).

Normal-hearing listeners gain important benefits from hearing with two ears, also called binaural hearing. One important advantage of binaural hearing is the possibility to determine from which direction sounds are coming (Middlebrooks 1991). The second advantage is the ability to hear sounds and understand speech in noisy environments, especially if sounds come from different directions (Bronkhorst 1988; Dirks 1969; MacKeith 1971). The following three factors contribute to the superior spatial hearing abilities in normal-hearing listeners:

1. *Binaural squelch*

The brain has a better representation of sounds with auditory input from two cochleas. Normal listeners are able to localize sounds in the horizontal plane (at ear level) with an accuracy of +/-14 degrees (Stevens 1936). When signals and noise come from different directions, the brain is able to separate them by comparing time, intensity and spectral differences between the two sides. The brain uses interaural differences in intensity and time between the two ears to determine the location of a sound source in the horizontal plane (Akeroyd 2006). The effect is that the brain is able to suppress signals that the listener does not wish to hear (Carhart 1965; Middlebrooks 1991). In normal hearing subjects, the squelch effect provides a gain of 2-4.9 dB in speech reception threshold (Bronkhorst 1988; Carhart 1965; MacKeith 1971).

2. Binaural summation

When identical signals are presented bilaterally, there is an advantage when hearing with both ears instead of just one alone. The brain uses binaural redundancy and binaural loudness summation to produce this advantage. Signal threshold is improved by 2–6 dB in the binaural listening condition over the monaural listening condition (Kamal 2012).

3. Head shadow effect

When listening to speech in noise, the head acts as an acoustic barrier that attenuates noise on the side contralateral to the signal. Therefore, when one ear is closer to the noise source, adding a second ear contralateral to the noise provides an ear with a better signal-to-noise ratio (SNR). The head shadow effect is purely geometric and does not require binaural processing by the brain. The head shadow effect is more pronounced at high frequencies, towards as much as 10-16 dB in frequencies above 1 kHz (Shaw 1974, Festen 1986).

During the last decades, cochlear implantation has become a widely accepted intervention in the treatment of patients with severe to profound bilateral sensorineural hearing loss. Cochlear implants (CIs) replace the function of a deaf human cochlea. A microphone, mounted to the skull of a patient, receives the sound waves. This microphone is connected to an electrode, which will be surgically implanted in the cochlea. The electrode replaces the function of the hair cells in the cochlea, generating frequency specific pulses resulting in signals from the auditory nerve to the auditory cortex.

Currently, it is standard clinical practice to provide a CI unilaterally in patients with bilateral profound to severe hearing loss. However, based upon the previous described well known advantages normal-hearing listeners derive from hearing with two ears, bilateral cochlear implantation is growing in popularity (Van Schoonhoven 2013). Although many patients with a unilateral CI achieve high levels of spoken word recognition when speech is presented in quiet, even the most successful users still experience difficulty in the presence of competing sounds and are poor at identifying the localization of sounds.

A recently published review showed a significant benefit of bilateral implantation in localization and speech perception in noise over unilateral cochlear implantation (Van Schoonhoven 2013). However, they highlighted the lack of high quality studies concerning the effectiveness of bilateral implantation, preferably RCTs. At this moment our study group is working on a RCT in which one group receives both CIs simultaneously and the other group receives the second implant after a 2-year delay. The results concerning the benefit of bilateral versus unilateral cochlear implantation will be presented later this year.

Up to now, patients with SSD are not considered candidates for cochlear implantation. In the Netherlands, current clinical practice for SSD consists of treatment with a CROSS, BAHA or no treatment. In CROSS, sound is routed from a satellite microphone on the deaf side into the hearing ear (Baguley 2006). If patients are not satisfied with CROSS therapy, placing of a BAHA is the next modality. A BAHA is a percutaneous titanium screw in the temporal bone of the skull, enabling direct transduction of vibrations to the contralateral cochlea (Hol 2004). Before a BAHA is implanted surgically, patients undergo a test period with a BAHA on a headband, simulating the effect, but avoiding the operation.

In 2006, Baguley et al. performed a meta-analysis and concluded that there are only four studies, with several methodological short-comings (i.e. study design, order effect, selection bias, underpowered studies), that compare CROSS to BAHA for patients with SSD. They did not find a significant improvement in auditory localization with either CROSS or BAHA (Baguley 2006). Speech discrimination in noise and subjective questionnaire measures of auditory abilities found BAHA favourable to CROSS and CROSS favourable to the unaided condition. In a more recent review, Bishop et al. stated that the shortcomings in the studies of this meta-analysis have not been overcome since (Bishop 2010). They concluded that the two approaches, both BAHA and CROSS, seemed to be effective in addressing the head shadow effect and restoring sound awareness to the affected side, but minimal to no benefits were found with regard to sound localization and improvement in speech perception.

The perceptive effects of binaural hearing can only be achieved with bilateral auditory input, which cannot be provided with either BAHA or CROSS. In both situations the contralateral cochlea is used to perceive sounds from the deaf ear instead of actual stimulation of the affected cochlea. Therefore patients with SSD still experience difficulties with binaural hearing. Approximately 55% of patients with SSD are not satisfied with current treatment modalities and prefer natural adaptation rather than implanting a BAHA (Desmet 2012). In 45% of these cases the inadequate speech understanding in noise is the main reason for rejection.

As quoted earlier in this paragraph, CIs restore the function of a deaf human cochlea; this way the old situation of binaural hearing may be recovered. Until now, only few non-randomised studies have been conducted on the topic of SSD and cochlear implantation. In unilaterally deaf patients ipsilateral cochlear implantation leads to significantly reduced error rates in spatial hearing tests with speakers located at different angles around patients compared to CROSS or BAHA error rates (Arndt 2010). Furthermore, the authors tested

several conditions for speech comprehension. With noise coming from the normal hearing side and the sound coming from the deaf side, speech comprehension is improved in the cochlear implant situation compared to BAHA, CROSS and the unaided situations 6 months after implantation (Arndt 2010).

Next to these audiological benefits, CIs entail other possible benefits for patients with SSD. Several studies found that cochlear implantation helps to reduce tinnitus (Arndt 2010; vd Heyning 2008, Távora-Vieira 2013). The paradigm is that tinnitus results from peripheral deafferentiation leading to a low stimulus state in the cortex. Once the afferent stimuli are back, which can be achieved with cochlear implantation, the tinnitus resolves (vd Heyning 2008).

Furthermore, both the audiological benefits and the absence of tinnitus result in a better quality of life (QoL) for patients with single-sided deafness. Távora-Vieira and colleagues assessed the QoL in their population with SSD patients using the Speech, Spatial and Qualities of Hearing Scale, consisting of subsections assessing speech understanding, spatial hearing and quality of hearing (Távora-Vieira 2013). They found a significant improvement on all three subscales for their study population three months after implantation. Arndt et al. showed significant improvement on both the Speech and Spatial sections of the SSQ after cochlear implantation (Arndt 2010).

While waiting for the results of ongoing RCTs evaluating the effectiveness of bilateral cochlear implantation in patients with severe to profound bilateral deafness, based on current knowledge bilateral cochlear implantation seems to be preferable compared to unilateral implantation when localization of sound and speech perception in noise are concerned (Van Schoonhoven 2013). The benefits of the natural binaural situation of hearing with two ears can only be achieved if both ears receive auditory input. For unilateral deaf patients, current treatment modalities with either CROS or BAHA do not deliver bilateral auditory signals and therefore patients with SSD experience difficulties with binaural hearing. For this reason, further prospective high quality research evaluating the treatment modalities for patients with SSD should be conducted.

2. OBJECTIVES

The main objective of our study is to evaluate the clinical outcome after cochlear implantation over standard health care therapy with either CROSS or BAHA in patients with SSD. As a

final point of interest, this study will also examine the cost utility of cochlear implantation for patients with single sided deafness.

3. STUDY DESIGN

The study design is illustrated in Figure 1 (next page). Patients participate after Informed Consent is obtained. In the dark blue squares of the figure, the different subsequent steps after inclusion are noted. The different time-points for follow-up are outlined in the yellow squares.

When subjects are selected, the following characteristics will be documented in a Case Report Form: gender, age, highest level of education, duration of unilateral deafness, anatomical and aetiological issues as well as psychological and motivational attributes.

After inclusion, subjects will be randomly allocated (see section 6.2 Randomisation, blinding and treatment allocation) to one of three treatment groups:

1. Group A: cochlear implantation
2. Group B: 6-week during test periods with first BAHA, then CROSS
3. Group C: 6-week during test periods with first CROSS, then BAHA

Subjects in group A, the cochlear implantation group, will receive a CI directly after randomisation. The device will be programmed 3-7 weeks after surgery, which is standard CI care.

Subjects in group B shall start with a 6 weeks during test period with a BAHA on a headband. After the 6 weeks, these subjects switch to another test period of 6 weeks with a CROSS. In group C, subjects start with a CROSS and after their first 6-week test period they switch to a test period with a BAHA on headband for 6 weeks. After these two test periods, three questionnaires will be filled out (SSQ, APHAB and GBI; see Section 6.1.2. Secondary study parameters/endpoints). The participants in groups B and C complete both test periods after 12 weeks. The reason for this cross-over is to control for bias caused by the order effect, which means that participants will judge the second modality influenced by their experience using the first modality. Since we have created two groups with the opposite sequence, the order effect is eliminated. After completing both test periods, subjects will be seen in our clinic and have to choose for further treatment with either CROSS, a definitive surgically implanted BAHA or no treatment (if they found none of the modalities helpful).

Figure 1: study design

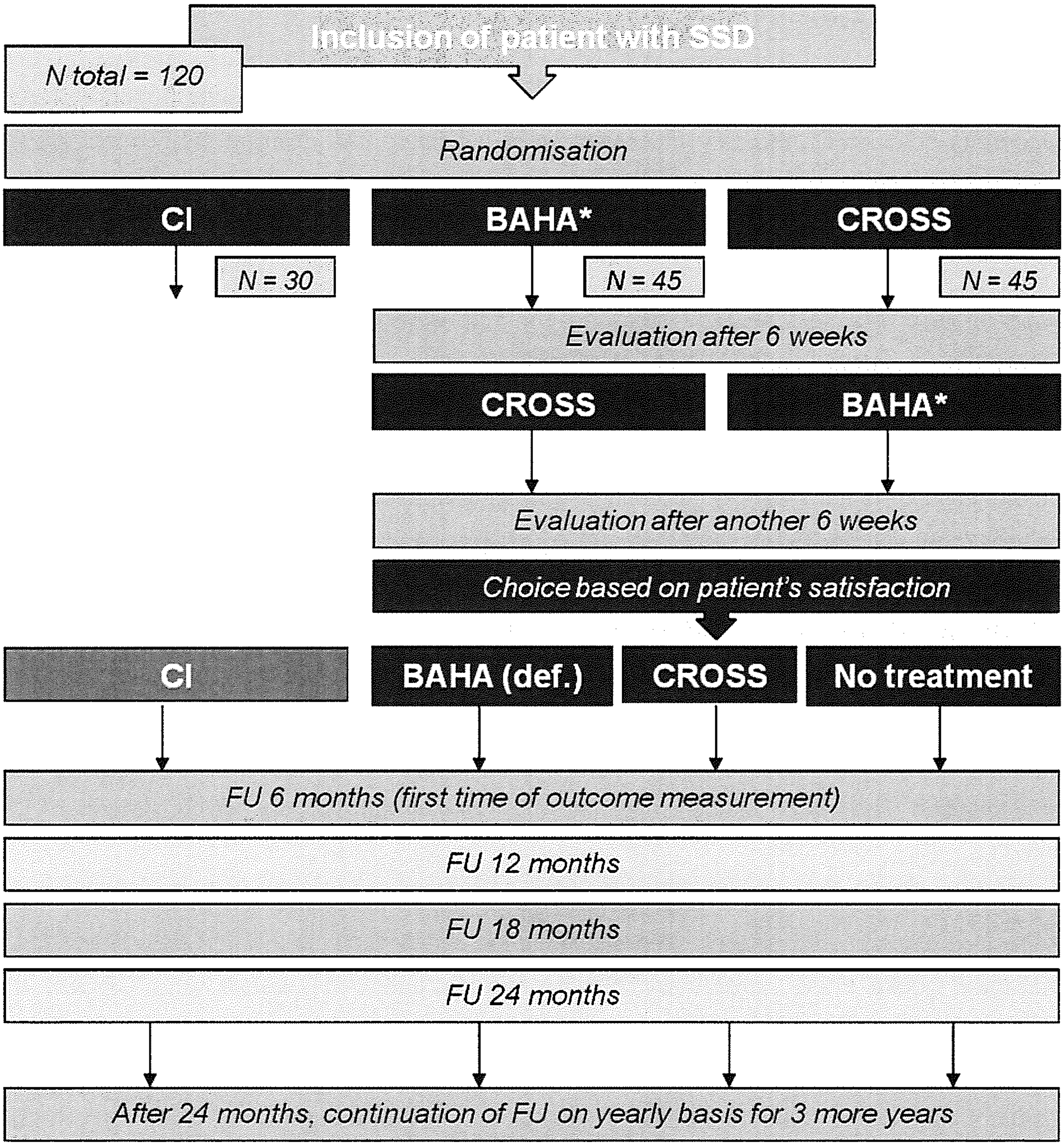

Legend: SSD: single-sided deafness, N: number of subjects, CI: cochlear implant; BAHA*: bone-anchored hearing aid on headband, BAHA (def): definitive surgically implanted BAHA, CROSS: contralateral routing of sound system, FU: follow-up.

In all groups the objective and subjective follow-up measurements shall take place at the same time-points. All questionnaires outlined in section 6.1.2. will be filled out.

During the first two years, subjects will be evaluated at 6, 12, 18 and 24 months after randomisation. During this period, the cost utility diary will be filled out every month. Afterwards, we will continue with follow-up on yearly basis for 3 more years (i.e. 36, 48 and 60 months post-randomisation). The cost utility diary will then be filled out every 3 months, to minimize subject burden.

A sample size of 30 subjects in group A and 45 subjects in group B and C each is designed to allow valid statistical assessment of cochlear implant benefit (see Section 4.4 Sample size calculation).

Patients with an onset of unilateral hearing loss between ≥3 months and ≤10 years before time of inclusion are eligible for inclusion (see also Section 4.2 Inclusion criteria). The reason for the three month interval after onset of deafness is that clinical practice nowadays is to first treat without hearing aids (for instance for sudden deafness with corticosteroids) and to await the natural course of the deafness. When the patient remains unilaterally deaf, he/she is eligible for inclusion in the study. When patients became deaf before this study was started, they are eligible for this study when the duration of deafness is ≤10 years. During this interval, adaptation to the monaural situation occurs and becomes irreversible thereafter. That is the reason why this cut-off point is established.

Subjects shall be randomised into one of the three groups using a strict randomisation process (see Section 6.2 Randomisation, blinding and treatment allocation). Subjects willing to participate must be informed that they can be assigned to either modality and accept to submit to this randomisation. It is only after the subject has signed their consent form that he/she is informed of the assigned group.

4. STUDY POPULATION

4.1 Population (base)

Subjects eligible for participation in the study must be adults with postlingual SSD due to cochlear damage. General practitioners and ENT-specialists in hospitals near Utrecht will be informed about the study and asked to inform and refer potential subjects with SSD to the ENT department of the UMC Utrecht.

4.2 Inclusion criteria

- Patients aged 18 or older.
- Acute onset of postlingual SSD, defined as onset unilateral hearing loss between ≥3 months and ≤10 years before time of inclusion.
- Hearing measurements:
  - Pure Tone Audiometry of the deaf ear, defined as thresholds of 70 dB or higher on frequencies 0,5 – 4.0 kHz (average).
  - Normal hearing on the contralateral ear, defined as pure tone audiometry thresholds of 30 dB or less on frequencies 0.5 – 4.0 kHz (average).
  - Air bone gap ≤10 dB.
- Normal function of middle ear (i.e. no acute middle ear infections or tympanic membrane perforations).
- Dutch language proficiency.
- Willingness and ability to participate in all scheduled procedures outlined in the protocol.
- General health allowing general anaesthesia for the potential surgical implantation of a CI or BAHA.
- Patients covered by the Dutch health insurance.
- Patients should agree to be implanted with a CI or BAHA.
- Informed consent understood, filled out and signed by patient.
- Patients are not allowed to participate in another ongoing research study related to SSD or cochlear implantation.

4.3 Exclusion criteria

- Previous experience with implanted BAHA or CI.
- Retrocochlear pathology.
- Abnormal cochlear anatomy in one or both ears (i.e. ossification).
- Disability which could interfere with the completion of the tests (i.e. psychiatric problems (e.g. auditory hallucinations could negatively affect performance on tests or questionnaires)), based on medical history as assessed by clinician and in electronical patient file.
- Severe comorbidity with an expected survival of less than five years, based on medical history as assessed by clinician and in electronical patient file.

4.4 Sample size calculation

To detect a clinically relevant difference of 5 dB SNR (standard deviation 5 dB) between the groups on the modified Plomp test, with an alpha of 0.05 and a power of 95%, 27 subjects

per group are needed. To be safe, a 10% margin is used to compensate for potential dropouts (e.g. non user cochlear implantees), resulting in 30 patients in the CI group. From recent literature we know that 55% of BAHA-on-headband users are not satisfied after their trial period and choose not to be implanted with a BAHA (Desmet 2012). Therefore, we must include more patients in groups B and C for compensation of the patients ending up in the 'no treatment' group. According to this proportion, 40 patients will end up with a BAHA. Patients not opting for a BAHA can choose for a CROSS. Based on many years of experience at our Audiologic Center, we expect ~60% to choose for CROSS. Thus, predicted numbers per group will be: CI (30), implanted BAHA (40), CROSS (30), 'no treatment' (20), leading to n = 45 for groups B and C. These numbers allow for a valid calculation of the clinically relevant difference on the modified Plomp test.

Based on figures from the UMC Utrecht (in Dutch: Bedrijfsbureau DHS), approximately 25-30 patients per year with SSD present at the ENT outpatient department. Together with patients from the peripheral area of the UMC Utrecht, we expect the inclusion of patients to last for 3-4 years after the inclusion of the first patient. We also expect single-sided deaf patients from other areas of the country to present at the ENT department at the UMC Utrecht.

5. TREATMENT OF SUBJECTS

5.1 Investigational product/treatment

Before study inclusion, subjects will receive standard workup for the diagnosis SSD. This ENT workup includes a clinical history and interview, otologic examination, audiological testing and a MRI-scan for auditory nerve evaluation. Additionally, to determine eligibility for cochlear implantation, a CT-scan will be made to visualize the anatomy of the mastoid. When the diagnosis SSD is sure, all inclusion criteria are met and anatomy permits cochlear implantation, inclusion and randomisation will take place.

Subjects will be asked to read and sign the Informed Consent form presented by the patient's ENT-surgeon before randomisation. This physician will fill in a Demographics Form to gather subject information.

Baseline evaluation for gathering study related data will take place locally at randomisation:

- Pure tone audiometry
- Speech audiometry (CVC [consonant-vowel-consonant] score in quiet)
- Questionnaires: SSQ, HUI3, GBI, APHAB, TTO, VAS, EQ5D, HADS, and Tinnitus questionnaires.

The patients will be asked to fill in a monthly costs diary starting on the first day after inclusion until the end of the second year of the study. From then on, they will complete the costs diary once per 3 months.

For all patients in group A (CI group) and those in group B or C preferring BAHA implantation preoperative medical evaluation will be carried out locally by the anaesthesiologist and includes a determination of general health status and suitability for surgery. Subjects in the CI group will also be evaluated by the center's Cochlear Implant Team, as is standard care for cochlear implantees. The standard surgical procedures developed for placement of the BAHA and the internal components of the CI will be followed. Specific procedures for implanting of the devices will be left to the discretion of the surgeon.

The first evaluation visit will occur approximately 3 to 7 weeks after surgery in the CI group to custom fit the processor software. It will be programmed for optimal speech perception, based on individual subject preferences.

In the BAHA and CROSS group patients will be evaluated after 6 and 12 weeks (see Figure 1). After finishing both test periods, the patient will be asked with which modality he or she wants to continue; either CROSS, BAHA or no treatment. When a patient chooses CROSS, he or she will be followed up by their own audiologist and returns to the UMC Utrecht for regular follow up sessions in this study. When a patient chooses a BAHA, a definitive BAHA will be implanted during an operation in the skull bone. Postoperative evaluation will take place according to regular BAHA-care procedures (e.g. mounting of abutment 6 weeks postoperatively when there is no skin reaction and software set up). Patients choosing no treatment will only be seen during the specified study follow up sessions.

In all treatment groups, standard evaluation sessions for collecting data will take place at 6, 12, 18 and 24 months after randomisation. After the first two years, subjects will be evaluated for data collection on yearly basis for three more years (36, 48 and 60 months). All objective evaluation measurements will take place at the UMC Utrecht to ensure that the data are gathered in an identical way. If considered necessary, extra sessions can exceptionally be added.

5.2 Use of co-intervention

Since we will only include patients with a normal hearing ear on the contralateral side, defined as thresholds of 30 dB or less on frequencies 0.5 – 4.0 kHz, patients shall not use a

contralateral hearing aid. The modalities nowadays used in clinical practice, are within the study protocol, so there is no co-intervention.

6. METHODS

6.1 Study parameters/endpoints

6.1.1 Main study parameter/endpoint

Performance on modified Plomp test with sentences in Dutch in the individual patient's best aided condition.

6.1.2 Secondary study parameters/endpoints

Objective

- Performance on Standard Dutch phoneme perception test (NvA-list)
- Performance on speech intelligibility with spatially separated sources
- Performance on Crescent of sounds test (Quentin Summerfield)

Subjective

- Self-reported benefits in everyday listening situations assessed with the Speech, Spatial and Qualities Hearing Scale (SSQ); 63 questions
- Quality-of-life (QoL) questionnaire score:
    - Health Utilities Index 3 (HUI3); 17 questions
    - Glasgow Benefit Inventory (GBI); 18 questions
    - Abbreviated Profile of Hearing Aid Benefit (APHAB); 24 questions
    - Time Trade Off (TTO); 1 question
    - Visual Analogue Scale (VAS); 2 questions
    - Euro-QoL 5D (EQ5D); 6 questions
    - Hospital Anxiety and Depression Scale (HADS); 14 questions
- Tinnitus questionnaires
    - Tinnitus Handicap Inventory (THI); 25 questions
    - Tinnitus Questionnaire (TQ); 52 questions
    - Tinnitus Burden Questionnaire; 12 questions
- Cost utility
    - Costs utility diaries on monthly basis (after 2 years: 3-monthly basis)

6.2 Randomisation, blinding and treatment allocation

120 subjects will randomly be assigned to one of the three study groups: 30 in the CI group, 45 in the first-BAHA group and 45 in the first-CROSS group. A website randomisation

program, developed by the Julius Center, UMC Utrecht (http://www.juliuscentrum.nl/random/) shall be used to divide the subjects into the groups.

The randomisation chart is established before the start of the study by an independent data manager. When a local executor recruits a subject, the physician can log on to the site and enter the subject's characteristics. A block randomisation model will be used to make sure that the ratio CI:CROSS:BAHA subjects will be about equal (30:45:45 = 2:3:3 per block). The physician will receive an allocation group number on his/her screen within a few seconds. It is only after the subject has signed the Informed Consent form that he/she is randomised and informed of the assigned group.

Logically, blinding is not possible since both patients and doctors will be able to see from the outside whether subjects have received a CI, BAHA or CROSS.

6.3 Study procedures

6.3.1 Objective

Modified Plomp test with sentences in Dutch

The Plomp sentence test is designed to determine a patient's ability to understand speech in a noisy environment. This provides a more comprehensive understanding of a person's listening capabilities in daily life than a speech intelligibility test in quiet only. The Plomp test outcome is the critical signal-to-noise-ratio (SNR) at which 50% of sentences is understood correctly. This level is called the Speech Reception Threshold in noise. Both sentences and noise are presented from the front at 70cm distance from the patient.

In the conventional Plomp test a response is considered correct when the presented sentence is repeated without any mistakes. A patient is tested in quiet first to check if the patient is able to perform the task. Although the Plomp test is commonly used in the clinic for patients with mild-moderate hearing loss, it has not been used routinely for cochlear implantees. A speech perception in noise test which is routinely used by a CI-team is a word perception in noise test. The reason for this is that a number of cochlear implant users will not be able to repeat sentences 100% correctly even if presented in quiet. However, in a word test patients cannot use their language ability, as they commonly do in understanding sentences. Therefore we have modified the scoring system of the Plomp test slightly. In the "modified Plomp test" we will be scoring the number of words repeated back correctly instead of the complete sentence. A sentence is considered to be repeated correctly when a subject repeats ≤2 words of the sentence incorrectly.

Test procedure:

- The sound level that corresponds to the patient's maximum CVC score in quiet (standard procedure) is the individual stimulus level that will be used for the modified Plomp test.
- The starting speech to noise level ratio (SNR) is set to +20 dB (noise 20dB less than individual sound level), at which, in most cases, less than two words per sentence should be repeated back incorrectly.
- The presentation of the noise starts 500 ms before the sentence and stops 500 ms after the sentence.
- If two or less words in a sentence are repeated back incorrectly, the SNR for the next sentence will be decreased by increasing the level of the noise, i.e. the task will be made more difficult. If more than two words are repeated back incorrectly, the SNR will be increased, i.e. the task will be made easier.
- Initially, the SNR will be changed in 10-dB steps. The step size will be reduced to 5 dB after one reversal and to 2.5 dB after the next reversal.
- This step size is used for the remainder of the sentences in the list. The average SNR used for the final ten sentences in the list is calculated and used as an estimate of the Speech Reception Threshold (SRT) in noise.

Speech intelligibility with spatially separated sources

This is a Speech Reception Threshold (SRT) test with spatially separated sources consisting of a target speech signal masked by an interfering signal. The speech material was taken from the sentences VU '98 CD (Versfeld 2000), recorded from a male and a female speaker. The spatial separation between the speech and the noise is introduced by positioning two loudspeakers at –60 and +60 degrees.

Crescent of sound (Quentin Summerfield)

This set of tests includes spatial and presentation level roving tests and will assess localization, lateralization, head shadow and squelch effect. A set of seven loudspeakers are controlled by a cluster of eight computers, creating situations representing real-life listening conditions and which may help to differentiate the abilities of subjects from the three different groups.

6.3.2 Subjective

Self reported benefits

Self-reported benefits in everyday listening situations will be assessed with the Speech, Spatial and Qualities Hearing Scale (SSQ) (Gatehouse 2004). The SSQ consists of three

scales that assess different domains of hearing: 1) The Speech Hearing subscale consists of 15 questions that assess the ability to separate speech from competing noise in a wide range of listening contexts, 2) The Spatial Hearing subscale consists of 17 questions that assess the ability to locate sound sources and their direction of movement, 3) The Quality of Hearing subscale consists of 19 questions that assess naturalness and clarity of sound sources. The subjects respond to each question using a rating scale that ranges from 0 (not at all) to 10 (excellent).

Quality-of-life questionnaires

- The *Health Utilities Index 3 (HUI3)* is a measure of general health status. It contains questions on eight domains: vision, hearing, speech, ambulation, dexterity, cognition, emotion and pain.
- *Time Trade-off (TTO):* comprises one question about how many years of the life patients are living at the moment, they would sacrifice for living with perfect hearing for the rest of their days.
- *Visual Analogue Scale (VAS):* comprises two 10cm scales on which patients can rate their hearing and health.
- *Euro-QoL5D:* is a measure of general health status. It contains questions on: mobility, self-care, daily activities, pain/complaints, anxiety/depression.
- The *Abbreviated Profile of Hearing Aid Benefit (APHAB)* is a 24 item, self assessment, disability based inventory to document the outcome of a hearing aid fitting and evaluate the fitting over time. The inventory yields scores on subscales for ease of communication, listening under reverberant conditions, listening in background noise and aversiveness of sound.
- The *Glasgow Benefit Inventory (GBI)* is a measure of patient benefit developed especially for otorhinolaryngological interventions (Robinson 1996). It is validated to measure outcomes on health status after otorhinolaryngological procedures. It measures QoL in three domains: social, general and physical. The domains score on a scale of -100 to 100 (minimum versus maximum benefit, respectively).
- *Hospital Anxiety and Depression Scale (HADS):* a fourteen item screening tool for anxiety and depression in non-psychiatric clinical populations.

Tinnitus Questionnaires

- *The Tinnitus Handicap Inventory (THI)* has 3 subscales: a functional subscale (11 items), an emotional subscale (9 items) and a catastrophic subscale (5 items).

- *The Tinnitus Questionnaire (TQ)* encompasses 52 questions on tinnitus related to: emotional distress, cognitive distress, intrusiveness, auditory perceptual difficulties, sleep disturbance and somatic complaints.
- *The Tinnitus Burden Questionnaire:* This questionnaire contains 12 questions on the severity and character of tinnitus described by subjects.

Cost utility diary:

Subjects are asked to keep a monthly diary involving physical complaints and costs regarding general health and their hearing aid. After two years, the subjects will complete the cost utility diary once per three months. Also this questionnaire will address sick leave and other labor productivity related issues. The questionnaires are developed for this study in consultation with a specialist in Economics in Health Care, affiliated with the Julius Center for Health Sciences and Primary Care, UMC Utrecht. The outcomes of this questionnaire will represent the *numerator* of the cost utility ratio.

The questionnaires will be filled out by patients starting on the day of surgery in the CI group and in group B and C after patients made their definitive choice for BAHA, CROSS or no treatment. Subjects are asked to specify complaints and/or costs in the diary. All subjects will be reminded on regular basis by email to keep filling in the diary.

The HUI3 and EQ5D questionnaire outcomes will be plotted in a graph in which the area under the curve will reflect Quality Adjusted Life Years (QALYs). Also, these data will produce the *denominator* of the cost utility ratio.

The patient can choose if he/she would like to fill in the subjective questionnaires digitally or in a paper version. This will be verified in the Informed Consent Form. The patient is able to change his/her preference during the study period. All questionnaires that cannot be transformed to digital questionnaires (SSQ, VAS, TTO, EQ5D, Tinnitus Burden Questionnaire) will be filled out by all patients on paper.

6.4 Withdrawal of individual subjects

Subjects can leave the study at any time for any reason if they wish to do so without any consequences. The principal investigator can decide to withdraw a subject from the study for urgent medical reasons.

6.4.1 Specific criteria for withdrawal

As before every surgery, the anaesthesiologist will evaluate the patient's health status when in Group A or when the patient has chosen for a definitive BAHA. In case the health status has worsened between inclusion and cochlear implant or BAHA surgery and the

anaesthesiologist advises not to continue, the patient will be excluded from further implantation.

6.5 Replacement for individual subjects after withdrawal

See also Section 4.4 Sample size calculation. To anticipate possible withdrawal 3 more subjects than needed according to the power calculation will be recruited in all groups. Therefore there will be no reason to replace subjects after withdrawal unless more subjects will withdraw than 3 per group.

6.6 Follow up of subjects withdrawn from treatment

During the study, each patient will stay in care of their own otorhinolaryngologist and audiologist, and subjects in group A also of their CI-team. In case a subject is withdrawn from the study he/she will continue with the standard medical treatment.

6.7 Premature termination of the study

Serious adverse events are not expected, but in case they do occur, each member of the research group has the right to terminate the study prematurely.

6.8 End of study

At the end of the study, all patients will be informed about the study outcomes. If this study shows CI is a superior treatment option for SSD, health insurance companies (Dutch: College voor Zorgverzekeraars [CvZ]) may change their reimbursement conditions. Patients from Groups B and C can opt for this new option via the ENT outpatient department. If this study shows BAHA or CROSS to be superior, reimbursement is already available in The Netherlands. They can also consult an ENT-specialist at the outpatient department for information on these treatment options. The procedure regarding their CI will be determined on an individual level.

7. SAFETY REPORTING

7.1 Section 10 WMO event

In accordance to section 10, subsection 1 of the WMO, the investigator will inform the subjects and the reviewing accredited METC if anything occurs, on the basis of which it appears that the disadvantages of participation may be significantly greater than was foreseen in the research proposal. The study will be suspended pending further review by the accredited METC, except insofar as suspension would jeopardise the subjects' health. The investigator will take care that all subjects are kept informed.

7.2 Adverse and serious adverse events

Adverse events (AEs) are defined as any undesirable experience occurring to a subject during a clinical trial, whether or not considered related to the investigational drug or intervention. All adverse events reported spontaneously by the subject or observed by the investigator or his staff will be recorded.
Besides the normal risks associated with surgery and general anaesthesia, AEs related to cochlear implantation or BAHA could be: implant failure, irritation, pain or infection and implant extrusion. Some specific AEs for cochlear implantation are complaints associated with electrical stimulation (i.e. tinnitus, facial nerve stimulation, dizziness) or uncomfortably loud sound sensation or no sound sensation due to failure of component parts.

A serious adverse event (SAE) is any untoward medical occurrence or effect that at any dose results in:

- death;
- is life threatening (at the time of the event);
- requires hospitalisation or prolongation of existing inpatients' hospitalisation;
- results in persistent or significant disability or incapacity;
- is a congenital anomaly or birth defect;
- is a new event of the trial likely to affect the safety of the subjects, such as an
- unexpected outcome of an adverse reaction, lack of efficacy of an investigational medicinal product used for the treatment of a life threatening disease, major safety finding from a newly completed animal study, etc.

All SAEs will be reported to the accredited METC that approved the protocol, according to the requirements of that METC.

7.3 Follow up of serious adverse events

In the case of implant failure, infection, extrusion or any other event resulting in the temporary non-use of the implant system, subjects will not be permanently excluded from the study, under the intention-to-treat-principle. Once the problem is solved, the subject will re-enter and continue the study for the amount of time left since he/she had not been operational with the device. A separate analysis for such a subject may be further conducted to control for possible biases.

All adverse events will be followed until they have abated, or until a stable situation has been reached. Depending on the event, follow up may require additional tests or medical procedures as indicated, and/or referral to the general physician or a medical specialist.

7.4 Monitoring

This study will be monitored according to regulations by the UMC Utrecht and the Dutch government. The monitoring plan will be outlined in a separate document.

8. STATISTICAL ANALYSIS

The aim of the study is to evaluate the clinical outcome after cochlear implantation, compared to BAHA, CROSS or unaided conditions, measured as speech perception in noise, sound localization, tinnitus and quality of life in patients with SSD. Also, a cost utility analysis will be performed (see section 6.3.2).

Overall, the study is an RCT, but subjects in group B and C run through the same two test periods within the first 3 months, afterwards they shall be treated with their modality of choice. As discussed previously, we expect 55% of patients in group B and C to choose for no treatment, we randomise more subjects in these groups (Desmet 2012). Thus equal comparison with the CI group will be possible during follow-up.

Major test intervals are the same in all study groups, namely 6, 12, 18, and 24 months after randomisation. After the first two years, subjects will be evaluated on yearly basis for three more years (36, 48 and 60 months).

The primary outcome will be the performance on the modified Plomp hearing test. The data are quantative and will be presented as continuous variables. Between-group mean differences, rate differences and rate ratios with 96% confidence intervals will be calculated. Differences between the three groups will be analysed using the Kruskal Wallis test.

The secondary outcomes will be the performances on the other auditory tests and the questionnaires (see Section 6.3). Analyses of between-group differences will be performed with Chi-square-tests for categorical outcomes and Kruskal Wallis tests for continuous outcomes. Within-subject comparisons will entail differences of mean values. These will be analysed using paired t-tests for continuous measures.

Missing values will be imputed using multiple imputation and all analyses will be performed on an intention-to-treat basis.

9. ETHICAL CONSIDERATIONS

9.1 Regulation Statement

The study will be conducted according to the principles of the Declaration of Helsinki (version 2013, Fortaleza) and in accordance with the Medical Research Involving Human Subjects Act (WMO).

9.2 Recruitment and consent

When a patient meets the criteria for inclusion in this study and for the potential cochlear implantation, he/she will be asked to participate by an otorhinolaryngologist. Like all patients eligible for cochlear implantation, the patients in this study will have several appointments with the members of the Cochlear Implant team (i.e. otorhinolaryngologist, audiologist, social worker etc.). The patients in group B and C will be evaluated in our clinic according to their choice for a BAHA, CROSS or no treatment.

There will be ample opportunity for the patient and his/her partner to consider participation and discuss their questions. The content of the study will be explained by the patient's otolaryngologist who will give the subject written patient information and the informed consent form. An independent physician is available for questions patients may still have afterwards.

9.3 Benefits and risks assessment, group relatedness

Compared to routine clinical practice in the Netherlands, the study requires that the subjects in group A undergo a cochlear implantation. This carries the usual risks associated with surgery in general and with some specific risks related to cochlear implantation or surgical placement of a BAHA. Cochlear implantation is already widely performed in bilaterally deaf patients. Theoretical benefits of cochlear implantation, with respect to sound localization, speech perception, tinnitus and quality of life were outlined in Section 1.2 Rationale; however, current evidence is based on low quality studies only.

For patients in groups B and C, there are no additional risks compared to standard practice. For BAHA surgery, standard risks of surgery are present (see also Section 7.2 Adverse and serious adverse events).

9.4 Compensation for injury

The sponsor/investigator has a liability insurance which is in accordance with article 7, subsection 6 of the WMO.

The sponsor (also) has an insurance which is in accordance with the legal requirements in the Netherlands (Article 7 WMO and the Measure regarding Compulsory Insurance for

Clinical Research in Humans of 23th June 2003). This insurance provides cover for damage to research subjects through injury or death caused by the study.

- € 450.000,-- (i.e. four hundred and fifty thousand Euro) for death or injury for each subject who participates in the Research, with a maximum of:
- € 3.500.000,-- (i.e. three million and five hundred thousand Euro) for the entire study.

The insurance applies to damage that becomes apparent during the study or within 4 years after the end of the study.

9.5 Incentives

All participants will receive compensation for travel expenses: €0.19 per kilometre.

10. ADMINISTRATIVE ASPECTS AND PUBLICATION

10.1 Handling and storage of data and documents

All data will be treated confidentially. The data will be encrypted by using a unique patient identification number. The analysis will be performed with these coded patient data. The key code will be safeguarded by the investigators. The paper data files will be stored in a locked room. The data will be stored on the investigator's computer as well, which is secured by a password and situated in a locked room. The subjects' general practitioner will be informed about the participation of the subject.

10.2 Amendments

Amendments are changes made to the research after a favourable opinion by the accredited METC has been given. All amendments will be notified to the METC that gave a favourable opinion. Non-substantial amendments will not be notified to the accredited METC and the competent authority, but will be recorded and filed.

10.3 Annual progress report

The investigator will submit a summary of the progress of the trial to the accredited METC once a year. Information will be provided on the date of inclusion of the first subject, numbers of subjects included and numbers of subjects that have completed the trial, serious adverse events/serious adverse reactions, other problems and amendments.

10.4 End of study report

The investigator will notify the accredited METC of the end of the study within a period of 8 weeks. The end of the study is defined as the last patient's last visit. In case the study is ended prematurely, the investigator will notify the accredited METC, including the reasons for the premature termination.

Within one year after the end of the study, the investigator will submit a final study report with the results of the study, including any publications/abstracts of the study, to the accredited METC.

10.5 Public disclosure and publication policy

The data from this study will be used for publication in peer-reviewed international journals. It will be part of a thesis on the clinical outcome of cochlear implantation in patients with SSD.

11. REFERENCES

- Akeroyd, MA. (2006) The psychoacoustics of binaural hearing. Int J Audiol 45:S25-S33.
- Arndt S, Aschendorff A, Laszig R et al. (2010) Comparison of pseudobinaural hearing to real binarual hearing rehabilitation after cochlear implantation in patients with unilateral deafness and tinnitus. Otol Neurotol 32:39-47
- Baguley DM, Bird J, Humphriss RL, Prevost AT. (2006) The evidence base for the application of contralateral bone anchored hearing aids in acquired unilateral sensorineural hearing loss in adults. Clin Otolaryngol 31:6-14
- Bishop CE, Eby TL. (2010) The current status of audiologic rehabilitation for profound unilateral sensorineural hearing loss. Laryngoscope 120:552-6
- Bronkhorst AW, Plomp R. (1988) The effect of head-induced interaural time and level differences on speech intelligibility in noise. J Acoust Soc Am 83:1508-1516.
- Carhart R. (1965) Monaural and binaural discrimination against competing sentences. Intern Audiol 4:5-10
- Desmet J, Bouzegta R, Hofkens A et al. (2012) Clinical need for a Baha trial in patients with single-sided sensorineural deafness. Analysis of a Baha database of 196 patients. Eur Arch Otolaryngol 269:799-605
- Dirks DD, Wilson RH. (1969) The effect of spatially separated sound sources on speech intelligibility. J Speech Hear Res 12:5-38.
- Festen JM and Plomp R. (1986) Speech-reception threshold in noise with one and two hearing aids. J Acoustic Soc Am 79:465-471
- Gatehouse S, Noble W. (2004) The speech, spatial and qualities of hearing scale (SSQ). Inter J Audio 43:85-99.
- Giolas T. (1994) Aural rehabilitation of adults with hearing impairment; in Katz J (ed): Handbook of Clinical Audiology, ed 4. Baltimore, Williams & Wilkins, pp 776-792.

- Hol MKS, Bosman AJ, Snik AFM, Mylanus EAM, Cremers CWRJ. (2004) Bone-anchored hearing aids in unilateral inner ear deafness: a study of 20 patients. Audiol Neuro-otol 9:274-281
- Kamal SM, Robinson AD, Diaz RC. (2012) Cochlear implantation in single-sided deafness for enhancement of sound localization and speech perception. Curr Opin Otolaryngol Head Neck Surg 20:393-7
- MacKeith NW, Coles RRA. (1971) Binaural advantages in hearing of speech. J Laryngol Otol 85:213-232.
- Middlebrooks JC, Green DM. (1991) Sound localization by human listeners. Ann Rev Psychol 42:135-159.
- Robinson K, Gatehouse S, Browning G et al. (1996) Measuring patient benefit from otorhinolaryngological surgery and therapy. Ann Otol Rhinol Laryngol 105:415-422
- Schoonhoven van J, Sparreboom M, Zanten van GA et al. (2013) The effectiveness of bilateral cochlear implants for severe-to-profound deafness in adults: a systematic review. Otol Neurotol 34:190-8
- Shaw EAG. (1974) Transformation of sound pressure level from the free field to the eardrum in the horizontal plane. J Acoustic Soc Am 56:1848-61
- Stevens SS, Newman EB. (1936) The localization of actual sources of sound. Am J Psychol 48:297-306.
- Távora-Vieira D, Marino R, Krishnaswamy J. (2013) Cochlear implantation for unilateral deafness with and without tinnitus: a case series. Laryngoscope 123:1251-5
- Heyning van de P, Vermeire K, Diebl M et al. (2008) Incapacitating unilateral tinnitus in single-sided deafness treated by cochlear implantation. Annals of Otol Rhinol Laryngol 117(9):645-652
- Vermeire K, Heyning van de P. (2009) Binaural hearing after cochlear implantation in subjects with unilateral sensorineural deafness and tinnitus. Audiol Neurotol 14:163-171
- Versfeld NJ, Daalder L, Festen JM et al. (2000) Method for the selection of sentence materials for efficient measurement of the speech reception threshold. J Acoust Soc Am 107:1671-84.
- Wie OB, Pripp AH, Tvete O. Unilateral deafness in adults: effects on communication and social interaction. Annals of Otol Rhinol Laryngol 119(11):772-781
- Woolf SH, Battista RN, Anderson GM et al & the Canadian Task Force on Periodic Health Examination. (1990) Assessing the clinical effectiveness of preventative manoeuvres: analytic principles and systematic methods in reviewing evidence and developing clinical practice recommendations. J of Clin Epidem 43:891-905
