# Supplementary material for: Short-term outcomes of cochlear implantation for single-sided deafness compared to bone conduction devices and contralateral routing of sound hearing aids—Results of a Randomised controlled trial (CINGLE-trial)
Source: PLoS One. 2021 Oct 13;16(10):e0257447. doi: 10.1371/journal.pone.0257447 (PMC8513831; doi:10.1371/journal.pone.0257447)
Supplement: S3 File — (PDF) [file pone.0257447.s003.pdf]

Supplemental digital content 1: Explanation of numbers per group at different follow-up moments

After randomisation

In the CI group (n = 29 patients randomised), one patient decided not to proceed with cochlear implantation because he found cochlear implantation too invasive, and withdrew from the study. Therefore, 28 patients were analyzed at baseline. One patient was lost to follow-up before the 3 months follow-up moment, and thus 27 patients were analyzed at 3 and 6 months follow-up. During the first 6 months of follow-up, there were no non-users.

In the 'first BCD, then CROS' group (n = 45 patients randomised), four patients decided to terminate the study before the end of the trial period (three patients were disappointed that they were not randomised to the CI group, one patient had his CI reimbursed by exception for his work with 'special needs' (police officer)). After the trial period 10 patients opted for the BCD, 17 patients chose for CROS, and 14 patients preferred no treatment.

In the 'first CROS, then BCD' group (n = 46 patients randomised), two patients decided to terminate the study before the end of the trial period (both lacked motivation to return for follow-up visits). After the trial period 15 patients opted for the BCD, 17 patients chose for CROS and 12 patients preferred no treatment.

After allocation of treatment

In the BCD group (n = 25 patients after trial period), one patient decided not to undergo surgery and terminated study participation. One patient was implanted, but due to recurrent skin infections around the BCD screw, it had to be removed and was not re-inserted. This patient was further analyzed in the No treatment group at 3 and 6 months follow-up. Hence, 25 patients were analyzed at baseline, and 23 patients were analyzed in the BCD group at 3 and 6 months follow-up.

In the CROS group (n = 34 patients after trial period), two patients did not use their CROS anymore after the 3 months follow-up moment, and were therefore measured without their device and analyzed in the No treatment group at 6 months. One patient was lost to follow-up after 3 months follow-up. Hence, 34

patients were analyzed in the CROS group at baseline and 3 months, and 31 patients were analyzed at 6 months follow-up.

In the No treatment group (n = 26 patients after trial period), one patient ended study participation before the 3 months follow-up moment because the follow-up visits were too demanding. As mentioned, one patient from the BCD group was analyzed in the No treatment group at 3 and 6 months. Another patient was lost to follow-up after 3 months. As mentioned, two patients from the CROS group were analyzed in the No treatment group at 6 months. So in total, 26 patients were analyzed in the No treatment group at baseline and 3 months, and 27 patients were analyzed at 6 months follow-up.
