# Supplementary material for: Short-term outcomes of cochlear implantation for single-sided deafness compared to bone conduction devices and contralateral routing of sound hearing aids—Results of a Randomised controlled trial (CINGLE-trial)
Source: PLoS One. 2021 Oct 13;16(10):e0257447. doi: 10.1371/journal.pone.0257447 (PMC8513831; doi:10.1371/journal.pone.0257447)
Supplement: S1 Table — (PDF) [file pone.0257447.s004.pdf]

Supplemental digital content 2:

Patient characteristics after allocation of treatment (i.e. CI activation, BCD implantation and CROS fitting).

	CI	BCD	CROS	No Treatment	Statistics
**Gender**					
Male:Female	13:14	8:15	21:13	10:16	ns[a]
**Age at inclusion (years)**					
Mean (SD)	52.5 (13.1)	55.6 (8.4)	52.1 (12.0)	52.1 (13.3)	ns[b]
**PTA_{be} (0.5 - 4 kHz) (dB)**					
Mean (SD)	15.0 (6.9)	14.2 (6.5)	16.0 (6.6)	15.2 (7.3)	ns[c]
Median [range]	15.0 [5.0-30.0]	12.5 [3.8-28.8]	16.3 [5.0-27.5]	17.5 [2.5-30.0]	
**Duration of deafness (years)**					
Mean (SD)	3.0 (2.9)	3.0 (3.1)	2.7 (3.0)	3.1 (2.9)	ns[c]
Median [range]	1.8 [0.3-10.0]	2.2 [0.3-10.0]	1.3 [0.3-10.0]	2.2 [0.3-10.0]	
**Etiology**					
Unknown	5	3	5	13	
Iatrogenic	1	0	0	1	
Sudden deafness	14	15	18	8	
Labyrinthitis	4	2	5	1	ns[a]
Infection *(not otherwise specified)*	0	0	2	1	
Ménière’s disease	3	2	3	0	
Traumatic	0	1	1	2	

Legend: There were no statistically significant differences between the groups.

Abbreviations:
CI = Cochlear Implant, BCD = Bone Conduction Device, CROS = Contralateral Routing of Sound hearing aid, ns = not significant ($p > 0.05$), SD = standard deviation, PTA_{be} = Pure Tone Average threshold of the best ear at 0.5, 1, 2, 4 kHz (dB).

[a] Fisher's Exact test. [b] One-way ANOVA. [c] Kruskal Wallis test.
