# Supplementary material for: Short-term outcomes of cochlear implantation for single-sided deafness compared to bone conduction devices and contralateral routing of sound hearing aids—Results of a Randomised controlled trial (CINGLE-trial)
Source: PLoS One. 2021 Oct 13;16(10):e0257447. doi: 10.1371/journal.pone.0257447 (PMC8513831; doi:10.1371/journal.pone.0257447)
Supplement: S2 Table — (PDF) [file pone.0257447.s005.pdf]

Supplemental digital content 3: data tables

Table 1: Speech perception in noise

	S_0N_0											
	**Baseline**				**3 months**				**6 months**			
	**CI**	**BCD**	**CROS**	**No Tr**	**CI**	**BCD**	**CROS**	**No Tr**	**CI**	**BCD**	**CROS**	**No Tr**
min	-5.0	-5.9	-5.9	-7.2	-6.9	-5.6	-5.9	-6.9	-5.9	-5.0	-6.6	-6.6
iqr 1	-4.1	-3.4	-4.3	-4.4	-5.0	-3.8	-3.8	-4.4	-5.5	-4.2	-3.9	-4.1
med	-2.8	-2.2	-3.6	-3.0	-4.4	-3.1	-3.4	-2.5	-4.1	-3.4	-3.4	-3.4
iqr 3	-1.8	-0.9	-2.5	-1.0	-3.0	-1.6	-3.1	-1.8	-2.8	-1.7	-2.5	-1.9
max	-0.6	12.8	-0.3	3.1	-0.9	0.9	-0.6	1.3	0.0	10.6	0.3	2.5
n	28	25	34	26	27	23	33	24	27	23	31	27

	$S_{pe}N_{be}$											
	**Baseline**				**3 months**				**6 months**			
	**CI**	**BCD**	**CROS**	**No Tr**	**CI**	**BCD**	**CROS**	**No Tr**	**CI**	**BCD**	**CROS**	**No Tr**
min	2.2	0.0	-0.9	0.3	-6.6	-5.0	-6.6	-0.6	-6.3	-6.3	-7.8	-0.3
iqr 1	3.4	3.8	2.9	3.8	-3.1	-2.2	-2.8	4.0	-3.8	-1.4	-2.3	3.6
med	4.7	5.0	4.4	4.7	-1.9	-0.9	-0.9	4.7	-2.2	-0.3	-1.3	5.0
iqr 3	6.6	6.3	5.6	6.2	-0.5	2.2	0.0	5.9	-1.4	1.6	0.8	7.0
max	7.8	19.4	10.6	10.6	2.2	5.6	4.7	10.3	1.9	3.8	2.2	11.6
n	28	25	34	26	27	23	33	24	27	23	31	27

	$S_{be}N_{pe}$											
	**Baseline**				**3 months**				**6 months**			
	**CI**	**BCD**	**CROS**	**No Tr**	**CI**	**BCD**	**CROS**	**No Tr**	**CI**	**BCD**	**CROS**	**No Tr**
min	-14.1	-14.7	-15.6	-15.0	-15.3	-12.5	-11.9	-14.4	-16.3	-11.9	-12.5	-14.1
iqr 1	-11.9	-11.6	-12.2	-11.8	-13.1	-8.1	-8.4	-12.5	-12.8	-9.1	-8.4	-12.8
med	-10.9	-10.0	-10.9	-9.7	-11.9	-7.2	-6.9	-10.8	-11.6	-7.8	-6.3	-10.6
iqr 3	-9.6	-8.4	-8.8	-7.9	-10.3	-5.9	-5.0	-9.1	-10.3	-5.8	-5.0	-8.6
max	-6.3	11.6	-5.0	-5.0	-5.9	4.4	2.2	-5.0	-6.6	10.3	0.9	-2.8
n	28	25	34	26	27	23	33	24	27	23	31	27

Legend:
Speech reception threshold in noise (SRTn) (in dB) at which patients were able to repeat 50% of the sentences correctly.
CI = cochlear implant, BCD = Bone Conduction Device, CROS = Contralateral Routing of Sound hearing aid, No Tr = No treatment

S_0N_0: speech and noise from the front, $S_{pe}N_{be}$ = speech presented to the poor ear, noise presented to the better ear, $S_{be}N_{pe}$ = speech presented to the better ear, noise presented to the poor ear.

Minimum (min) and maximum (max) values are presented, as well as first and third quartiles (iqr 1 and iqr 3, respectively), medians (med) and number of measurements (n).

Table 2: Sound localization

	15°											
	Baseline				3 months				6 months			
	CI	BCD	CROS	No Tr	CI	BCD	CROS	No Tr	CI	BCD	CROS	No Tr
min	13.3	10.0	10.0	13.3	16.7	13.3	10.0	16.7	23.3	16.7	6.7	10.0
iqr 1	20.0	20.0	20.0	20.0	36.7	21.7	23.3	20.0	43.3	18.3	20.0	20.0
med	26.7	26.7	23.3	21.7	43.3	26.7	26.7	23.3	50.0	26.7	26.7	20.0
iqr 3	30.0	33.3	32.5	26.7	46.7	35.0	36.7	30.0	53.3	31.7	35.0	33.3
max	93.3	63.3	93.3	76.7	70.0	56.7	100.0	76.7	80.0	56.7	96.7	73.3
n	28	25	34	26	27	23	33	24	27	23	31	27

	30°											
	Baseline				3 months				6 months			
	CI	BCD	CROS	No Tr	CI	BCD	CROS	No Tr	CI	BCD	CROS	No Tr
min	20.0	13.3	16.7	13.3	23.3	20.0	16.7	20.0	40.0	20.0	16.7	13.3
iqr 1	25.8	26.7	26.7	20.0	50.0	25.0	26.7	23.3	53.3	26.7	28.3	23.3
med	36.7	33.3	35.0	31.7	60.0	33.3	30.0	30.0	63.3	33.3	36.7	33.3
iqr 3	50.8	60.0	53.3	39.2	73.3	40.0	46.7	54.2	75.0	40.0	48.3	46.7
max	100.0	93.3	100.0	76.7	83.3	86.7	100.0	70.0	90.0	96.7	96.7	86.7
n	28	25	34	26	27	23	33	24	27	23	31	27

	60°											
	Baseline				3 months				6 months			
	CI	BCD	CROS	No Tr	CI	BCD	CROS	No Tr	CI	BCD	CROS	No Tr
min	30.0	30.0	23.3	30.0	40.0	33.3	30.0	26.7	60.0	26.7	26.7	20.0
iqr 1	48.3	46.7	45.0	36.7	78.3	43.3	43.3	33.3	85.0	53.3	40.0	36.7
med	65.0	66.7	66.7	55.0	86.7	56.7	63.3	56.7	93.3	60.0	66.7	56.7
iqr 3	77.5	96.7	79.2	73.3	96.7	70.0	73.3	80.8	100.0	76.7	73.3	75.0
max	100.0	100.0	100.0	100.0	100.0	100.0	100.0	100.0	100.0	100.0	100.0	100.0
n	28	25	34	26	27	23	33	24	27	23	31	27

Legend:
Values in percentage correct score (%).
CI = cochlear implant, BCD = Bone Conduction Device, CROS = Contralateral Routing of Sound hearing aid, No Tr = No treatment

Configurations:
15º: 5 loudspeakers separated by an angle of 15º (-30º, -15º, 0º, +15º, and +30º).
30º: 5 loudspeakers separated by an angle of 30º (-60º, -30º, 0º, +30º, and +60º).
60º: 3 loudspeakers separated by an angle of 60º (-60º, 0º, and +60º).

Minimum (min) and maximum (max) values are presented, as well as first and third quartiles (iqr 1 and iqr 3, respectively), medians (med) and number of measurements (n).

Table 3: Questionnaires on tinnitus burden

Table 3a: TQ

	TQ											
	Baseline				3 months				6 months			
	CI	BCD	CROS	No Tr	CI	BCD	CROS	No Tr	CI	BCD	CROS	No Tr
min	5.0	2.0	1.0	1.0	1.0	1.0	3.0	11.0	1.0	0.0	1.0	1.0
iqr 1	13.0	12.3	14.0	22.0	3.8	9.8	14.0	25.0	6.0	6.0	8.8	22.0
med	25.5	24.0	21.0	30.0	10.5	17.5	19.0	29.0	9.0	12.0	17.0	26.0
iqr 3	42.5	42.3	39.0	46.5	19.0	34.8	29.0	34.0	16.0	26.0	34.5	30.0
max	59.0	70.0	70.0	74.0	57.0	54.0	59.0	76.0	63.0	55.0	59.0	70.0
n	26	22	33	24	20	18	31	21	21	21	28	24

Table 3b: THI

	THI											
	Baseline				3 months				6 months			
	CI	BCD	CROS	No Tr	CI	BCD	CROS	No Tr	CI	BCD	CROS	No Tr
min	2.0	0.0	0.0	0.0	0.0	0.0	0.0	10.0	0.0	0.0	0.0	0.0
iqr 1	14.0	11.0	14.0	18.0	3.5	8.0	14.0	18.0	4.0	0.0	10.0	21.5
med	27.0	21.0	24.0	26.0	7.0	14.0	18.0	30.0	8.0	10.0	23.0	25.0
iqr 3	45.5	37.5	42.0	42.0	29.0	28.0	28.0	42.0	12.0	30.0	36.5	38.0
max	74.0	84.0	88.0	88.0	78.0	64.0	70.0	92.0	78.0	70.0	66.0	94.0
n	26	22	33	24	20	18	31	21	21	21	28	24

Legend:
Table 3a: results on the TQ. Possible range of score 0.0-80.0.
Table 3b: results on the THI. Possible range of score 0.0-100.0.

CI = cochlear implant, BCD = Bone Conduction Device, CROS = Contralateral Routing of Sound hearing aid, No Tr = No treatment

At baseline and at 3 and 6 months follow-up, patients indicated whether they had tinnitus or not. Patients with no tinnitus, did not complete the questionnaires on tinnitus burden.

Minimum (min) and maximum (max) values are presented, as well as first and third quartiles (iqr 1 and iqr 3, respectively), medians (med) and number of measurements (n).

Table 4: SSQ questionnaire

	Speech											
	Baseline				3 months				6 months			
	CI	BCD	CROS	No Tr	CI	BCD	CROS	No Tr	CI	BCD	CROS	No Tr
min	4.3	14.1	17.7	14.1	24.2	22.9	25.0	5.8	24.0	24.9	22.2	11.4
iqr 1	30.5	20.7	33.4	24.7	55.6	39.3	40.8	28.1	53.6	35.7	41.4	30.0
med	36.1	31.8	42.5	35.6	61.4	53.2	49.3	36.9	61.3	49.4	48.5	37.4
iqr 3	44.9	44.1	47.5	45.7	69.9	63.8	57.9	49.6	70.1	60.0	58.9	46.4
max	75.0	60.0	60.6	64.6	86.9	78.5	79.6	61.9	82.8	78.1	78.4	60.7
n	28	25	34	26	27	23	33	23	27	23	31	27

	Spatial											
	Baseline				3 months				6 months			
	CI	BCD	CROS	No Tr	CI	BCD	CROS	No Tr	CI	BCD	CROS	No Tr
min	4.1	7.5	7.9	6.5	5.6	15.3	8.6	6.9	14.1	12.5	13.5	8.2
iqr 1	14.9	17.6	18.7	16.0	38.1	26.9	27.6	14.4	42.1	23.4	21.6	15.7
med	22.9	21.8	25.9	28.2	51.3	36.2	39.3	30.0	55.9	37.4	38.4	26.3
iqr 3	35.6	30.5	40.0	41.6	64.7	53.5	52.4	40.8	65.3	56.8	44.8	40.2
max	44.2	52.9	71.8	76.6	74.0	69.1	77.0	83.8	77.4	76.9	73.5	71.8
n	28	25	34	26	27	23	33	23	27	23	31	27

	Qualities of hearing											
	Baseline				3 months				6 months			
	CI	BCD	CROS	No Tr	CI	BCD	CROS	No Tr	CI	BCD	CROS	No Tr
min	32.8	21.2	41.9	25.7	33.1	42.4	47.6	21.9	44.4	23.7	46.1	28.1
iqr 1	52.2	46.1	57.1	48.9	60.0	54.2	61.3	46.6	60.5	48.9	59.9	51.3
med	58.5	54.9	64.4	61.6	68.9	66.5	68.9	54.8	68.3	60.6	65.1	59.6
iqr 3	66.3	65.3	71.2	65.6	73.4	73.5	74.7	68.6	73.8	71.6	73.5	67.3
max	75.5	80.8	80.8	76.9	81.7	90.9	88.9	77.8	83.2	94.7	85.6	79.1
n	28	25	34	26	27	23	33	23	27	23	31	27

Legend:
Results on the SSQ questionnaire per subscale. Possible range of score 0.0-100.0. The responses were given on a visual analogue scale; a higher score reflects a better performance.

CI = cochlear implant, BCD = Bone Conduction Device, CROS = Contralateral Routing of Sound hearing aid, No Tr = No treatment

Minimum (min) and maximum (max) values are presented, as well as first and third quartiles (iqr 1 and iqr 3, respectively), medians (med) and number of measurements (n).

Table 5: APHAB questionnaire

	EC											
	Baseline				3 months				6 months			
	CI	BCD	CROS	No Tr	CI	BCD	CROS	No Tr	CI	BCD	CROS	No Tr
min	0.11	0.07	0.09	0.08	0.05	0.01	0.05	0.11	0.05	0.01	0.07	0.12
iqr 1	0.24	0.23	0.21	0.18	0.10	0.16	0.18	0.18	0.12	0.14	0.17	0.23
med	0.33	0.38	0.27	0.33	0.15	0.26	0.21	0.28	0.15	0.26	0.25	0.29
iqr 3	0.50	0.54	0.40	0.45	0.25	0.31	0.29	0.46	0.23	0.31	0.29	0.46
max	0.56	0.93	0.62	0.83	0.52	0.40	0.50	0.79	0.37	0.40	0.56	0.79
n	28	25	34	26	24	20	32	24	26	22	30	27

	BN											
	Baseline				3 months				6 months			
	CI	BCD	CROS	No Tr	CI	BCD	CROS	No Tr	CI	BCD	CROS	No Tr
min	0.48	0.35	0.39	0.35	0.07	0.27	0.19	0.38	0.07	0.18	0.12	0.31
iqr 1	0.65	0.64	0.62	0.59	0.25	0.39	0.46	0.56	0.28	0.42	0.46	0.57
med	0.74	0.73	0.72	0.71	0.46	0.58	0.59	0.71	0.41	0.52	0.59	0.77
iqr 3	0.77	0.85	0.84	0.79	0.59	0.68	0.68	0.77	0.48	0.63	0.76	0.84
max	0.99	0.95	0.93	0.95	0.83	0.89	0.89	0.99	0.83	0.91	0.87	0.97
n	28	25	34	26	24	20	32	24	26	22	30	27

	RV											
	Baseline				3 months				6 months			
	CI	BCD	CROS	No Tr	CI	BCD	CROS	No Tr	CI	BCD	CROS	No Tr
min	0.29	0.10	0.13	0.08	0.12	0.16	0.09	0.12	0.05	0.01	0.09	0.16
iqr 1	0.43	0.46	0.44	0.42	0.18	0.29	0.24	0.34	0.18	0.30	0.29	0.42
med	0.62	0.60	0.55	0.53	0.29	0.38	0.30	0.52	0.28	0.39	0.33	0.52
iqr 3	0.67	0.75	0.65	0.70	0.42	0.57	0.42	0.61	0.35	0.53	0.45	0.68
max	0.87	0.95	0.91	0.91	0.69	0.69	0.69	0.91	0.54	0.75	0.71	0.89
n	28	25	34	26	24	20	32	24	26	22	30	27

	AS											
	Baseline				3 months				6 months			
	CI	BCD	CROS	No Tr	CI	BCD	CROS	No Tr	CI	BCD	CROS	No Tr
min	0.11	0.03	0.07	0.08	0.03	0.05	0.03	0.11	0.08	0.03	0.07	0.11
iqr 1	0.37	0.31	0.36	0.44	0.18	0.23	0.29	0.34	0.17	0.25	0.37	0.30
med	0.54	0.50	0.50	0.60	0.35	0.38	0.42	0.52	0.31	0.36	0.46	0.62
iqr 3	0.65	0.64	0.61	0.74	0.52	0.54	0.50	0.68	0.46	0.48	0.59	0.73
max	0.87	0.79	0.83	0.97	0.83	0.70	0.69	0.97	0.85	0.81	0.81	0.97
n	28	25	34	26	24	20	32	24	26	22	30	27

Legend:
Results on the APHAB questionnaire per subscale (ease of communication (EC), listening under reverberant conditions (RV), listening in background noise (BN), and aversiveness of sounds (AS)). Possible range of score 0.0-100.0 per subscale. A lower score reflects less problems in daily life.

CI = cochlear implant, BCD = Bone Conduction Device, CROS = Contralateral Routing of Sound hearing aid, No Tr = No treatment

Minimum (min) and maximum (max) values are presented, as well as first and third quartiles (iqr 1 and iqr 3, respectively), medians (med) and number of measurements (n).

Table 6: GBI

	General					
	3 months			6 months		
	CI	BCD	CROS	CI	BCD	CROS
min	-25.00	0.00	-18.18	-75.00	-8.33	-29.17
iqr 1	14.58	8.33	4.17	10.80	8.33	10.80
med	29.17	20.83	20.83	25.00	20.83	20.83
iqr 3	41.67	33.33	33.33	33.33	29.17	29.17
max	66.67	54.17	54.17	79.17	77.27	58.33
n	27	23	31	27	23	31

	Social support					
	3 months			6 months		
	CI	BCD	CROS	CI	BCD	CROS
min	0.00	-33.33	-16.67	0.00	-16.67	-50.00
iqr 1	0.00	0.00	0.00	0.00	0.00	0.00
med	0.00	0.00	0.00	0.00	0.00	0.00
iqr 3	25.00	0.00	0.00	8.33	0.00	0.00
max	50.00	33.33	16.67	33.33	33.33	33.33
n	27	23	31	27	23	31

	Physical health					
	3 months			6 months		
	CI	BCD	CROS	CI	BCD	CROS
min	-50.00	-33.33	-33.33	-33.33	-33.33	-50.00
iqr 1	0.00	0.00	0.00	0.00	0.00	0.00
med	0.00	0.00	0.00	0.00	0.00	0.00
iqr 3	0.00	0.00	0.00	0.00	0.00	0.00
max	16.67	0.00	16.67	16.67	16.67	0.00
n	27	23	31	27	23	31

Legend:
Results on the Glasgow Benefit Inventory (GBI) per subscale (general, social support, physical health). Possible range of score -100.0 to 100.0 per subscale. A higher score reflects more benefit from the intervention, a negative score reflects disadvantage from the intervention. The only statistically significant difference between-groups was the difference between the CI and CROS groups at 3 months follow-up (p = 0.018).

CI = cochlear implant, BCD = Bone Conduction Device, CROS = Contralateral Routing of Sound hearing aid

Minimum (min) and maximum (max) values are presented, as well as first and third quartiles (iqr 1 and iqr 3, respectively), medians (med) and number of measurements (n).
