# Supplementary material for: Short-term outcomes of cochlear implantation for single-sided deafness compared to bone conduction devices and contralateral routing of sound hearing aids—Results of a Randomised controlled trial (CINGLE-trial)
Source: PLoS One. 2021 Oct 13;16(10):e0257447. doi: 10.1371/journal.pone.0257447 (PMC8513831; doi:10.1371/journal.pone.0257447)
Supplement: Attachment — Submitted filename: Reply to Reviewers_Jan 21.docx [file pone.0257447.s006.docx]

**Reply to Reviewers letter PONE-D-20-34585**

Original comments

Reply to Reviewers by authors

>>First of all, we would like to thank the editor and reviewer for their detailed feedback and review of our manuscript. In this letter we respond (in blue) to each point raised by the editor and reviewer and made changes in the manuscript accordingly.

**Editor**

Please pay careful attention to the review comments, and also please address the following issues.

Why would you use blocked randomization, given its well known fatal flaws?

>>The main reason to use block randomisation was to achieve balanced allocation of participants to the three groups over time. We agree that a well-known disadvantage of block randomization is that the allocation of participants may be predictable and result in selection bias when the study groups are unmasked. However, in this trial we did not (could not) blind researchers or patients given for the intervention. Therefore, this disadvantage of block randomization was not applicable to our trial.

Clearly the data are not normally distributed. That could have been determined by appeal to the fact that no data are normally distributed. Why would you feel the need to test to arrive at this conclusion?

>>Our statistical analysis plan was designed to first check if data were normally distributed, so we could know which type of tests to use (parametric or non-parametric). We agree that our data are clearly not normally distributed; however, there is no harm to check this statistically. For clarity for the readers, we removed the first sentence of the section ‘statistical analysis’ for easier reading and more clarity.

Where are the p-values in Table 1?

>>Since patients were randomly distributed across groups, statistical comparison between baseline characteristics is not advocated. In short, if there would be a statistically significant difference, it would be based on chance by definition (since randomised allocation occurred). See for example the Editorial: *Statistical testing for baseline differences between randomised groups is not meaningful* via <https://www.nature.com/articles/s41393-018-0203-y>)

==============================

If you would like to make changes to your financial disclosure, please include your updated statement in your cover letter.

1. Please ensure that your manuscript meets PLOS ONE's style requirements, including those for file naming.

>>We changed the layout of the manuscript, (figure) legends and supporting information according to the PLOS One style requirements.

2. Please ensure that all deviations from the original approved protocol are discussed and justified in the manuscript text. In particular, please provide a justification why some questionnaire used to assess quality of life as a secondary outcome are not detailed in the Methodology.

>>We explained deviations from the original protocol in the Introduction (lines 131-132), where we specified that some secondary outcomes will be discussed in a separate manuscript that will follow. Moreover, in the Materials and Methods (line 142-144) we detailed the addition of the extra follow-up moment 3 months after device activation.

Furthermore, please provide additional details to the inclusion criteria within the Manuscript text.

>>We added information on the inclusion criteria, so it now equals the exact text from the original research protocol and published study protocol manuscript (lines 154-159).

And finally please provide clarification regarding the discrepancy in the start date between the manuscript and the clinical trial registry.

>>Thank you for this remark. The start date in the clinical trial register was incorrect. We updated the info in the clinical trial register (see <https://www.trialregister.nl/trial/4457>), and now the start date is the same in the clinical trial register and in the manuscript (start date/first inclusion July 15^th^, 2014).

3. Thank you for submitting your clinical trial to PLOS ONE and for providing the name of the registry and the registration number. The information in the registry entry suggests that your trial was registered after patient recruitment began. PLOS ONE strongly encourages authors to register all trials before recruiting the first participant in a study.

>>Please see the info online (<https://www.trialregister.nl/trial/4457>). We registered our trial online (May 6^th^, 2014) which was before inclusion of the first patient (July 14^th^, 2014).

4. Thank you for stating the following in the Financial Disclosure section:

'This study is partly funded by Cochlear Ltd. as an unrestricted research grant. By research contract, Cochlear Ltd. did not have influence on the study design, data collection, analysis, data interpretation, and publication. The funders had no role in study design, data collection and analysis, decision to publish, or preparation of the manuscript.'

We note that you received funding from a commercial source: Cochlear Ltd

a. Please provide an amended Competing Interests Statement that explicitly states this commercial funder, along with any other [relevant declarations](about:blank) relating to employment, consultancy, patents, products in development, marketed products, etc.

>>We amended the Competing Interests Statement in the manuscript (page 2).

Within this Competing Interests Statement, please confirm that this does not alter your adherence to all PLOS ONE policies on sharing data and materials by including the following statement: "This does not alter our adherence to PLOS ONE policies on sharing data and materials.” (as detailed online in our guide for authors [http://journals.plos.org/plosone/s/competing-interests](about:blank)).  If there are restrictions on sharing of data and/or materials, please state these. Please note that we cannot proceed with consideration of your article until this information has been declared.

b. Please include your amended Competing Interests Statement within your cover letter. We will change the online submission form on your behalf.

>>We amended the Competing Interests Statement also in the new cover letter.

**5. Review Comments to the Author**

Please use the space provided to explain your answers to the questions above. You may also include additional comments for the author, including concerns about dual publication, research ethics, or publication ethics. (Please upload your review as an attachment if it exceeds 20,000 characters)

**Reviewer #1**: The authors aimed to compare different treatments (CI, BCD, CROS and no treatment) to support patients with single-side deafness. The trial is described as randomized controlled trial where the description suggests that it is a patient preference design.

From a methodological point of view, I have some difficulties with the analysis and subsequent conclusion.

First, patients were randomised to CI or to one of the treatment sequences BCF followed by CROS or CROS followed by BCF as in a cross-over design. After this “randomized” run-in period, patients from the cross-over groups were treated based on their preference with BCD, CROS or no treatment. Patients treated initially with CI were further treated with CI without having a choice. These options lead to seven different groups, namely CI; BCF based on BCF/CROS; CROS based on BCF/CROS; No treatment based on BCF/CROS; BCF based on CROS/BCF; CROS based on CROS/BCF and No treatment based on CROS/BCF but were analysed as four (CI, BCF, CROS and no treatment). This approach ignores the effects from the pre-treatment, causes confounding and thus implies bias in the analysis. Comparisons using the non-randomised and confounded groups are unreliable because of the presence of uncontrolled confounders.

>>We aimed to compare CI to the current clinical practice in The Netherlands (lines 186-188), which consists of a trial period with BCD and CROS. Patients were randomly assigned to CI or to the ‘clinical practice’ groups. We agree with the reviewer that the order in which the ‘current clinical practice’ devices are tested by participants may be of influence on their decision which they prefer. To correct for this ‘order effect’ (line 179), we designed two groups: ‘first BCD, then CROS’, and ‘first CROS, then BCD’, and both then lead to the final groups: BCD, CROS or No treatment (like in current clinical practice).

It may indeed be interesting, as the reviewer suggests, to analyse differences between patients opting for BCD after having tested the BCD first compared to having the BCD tested second. As detailed in the study protocol (S1_File), a detailed analysis will follow in a later manuscript. For clarity of the reader, we also added this information to the Discussion section (lines 629-631).

One possible option would be to use the randomisation status as a covariate. In general, a patient preference trial should be treated as an observational study with known confounding factors adjusted for in the analysis. This also means that the STROBE checklist is more suitable than CONSORT.

>>Thank you for your comment. Since we used a randomized study design, we used the CONSORT Statement, which is suitable to assess the randomization (e.g. sequence generation) and allocation process. None of the study types that the STROBE checklist was designed for contains this information. Nevertheless, it would be interesting to use the randomisation status as a covariate in the detailed analysis of the trial period.

The analysis performed by the authors includes comparison between to measurements moments within one group or between two groups at the same follow-up moment. But what is in fact of interest is the comparison of changes between groups. I would suggest to adapt the analysis accordingly to the above mentioned aspects.

>>Thank you for this suggestion. For all outcomes, the test/questionnaire median results from baseline and the 3 and 6 months follow-up moments are displayed in Figures 3-6. These results were statistically compared between groups, as was detailed in the data analysis plan. In these figures, readers can also see the changes between groups. All exact medians, interquartile ranges and minimum/maximum values are provided in S5_Tables. In this manuscript, we did not statistically compare the differences/changes in follow-up moments between groups.

Moreover, in the protocol is stated, that missing values will be imputed using a multiple imputation approach and all analyses will be performed on an intention-to-treat basis. Since a protocol is a binding document, the analysis should follow the agreed details.

>>We thank the reviewer for this remark. Given the very low proportion of missing data in our study, we chose not to use multiple imputation. When designing the study, we took a larger proportion of missing data into account, for which we aimed to use multiple imputation. Due to the nature of our interventions, subject data were analysed ‘as treated’. To make it clear that we deviated from the original protocol, we added this information to the ‘Statistical analysis’ section.

For the same reason, a justification for the evaluation of the 3 months measurements should be provided, because in the protocol planned evaluation starts at 6 months after randomisation.

>>It is correct that we added the 3 month follow-up moment after first approval of the study protocol. However, we added this follow-up moment before inclusion of the first patient. In lines 142-145 we detailed this deviation from the original protocol.
