# Supplementary material for: Short-term outcomes of cochlear implantation for single-sided deafness compared to bone conduction devices and contralateral routing of sound hearing aids—Results of a Randomised controlled trial (CINGLE-trial)
Source: PLoS One. 2021 Oct 13;16(10):e0257447. doi: 10.1371/journal.pone.0257447 (PMC8513831; doi:10.1371/journal.pone.0257447)
Supplement: Attachment — Submitted filename: Reply to Reviewers_Mar 12.docx [file pone.0257447.s007.docx]

**Reply to Reviewers letter PONE-D-20-34585 R1**

Original comments and previous reply by authors

Current reply to editor by authors

**Editor**
I am repeating the earlier comments, and your replies to them, for ease of reference.
Please pay careful attention to the review comments, and also please address the following issues.

Why would you use blocked randomization, given its well known fatal flaws?
>>The main reason to use block randomisation was to achieve balanced allocation of participants to the three groups over time. We agree that a well-known disadvantage of block randomization is that the allocation of participants may be predictable and result in selection bias when the study groups are unmasked. However, in this trial we did not (could not) blind researchers or patients given for the intervention. Therefore, this disadvantage of block randomization was not applicable to our trial.
You have that completely wrong. The problem with permuted blocks is especially acute in unmasked trials. How would you possibly think that unmasking would somehow mitigate or eliminate this problem? Obviously, you cannot go back now and correct your mistake, but you can admit that you made a rather serious mistake, you can agree to randomize better in the future, and you can test for the extent to which your mistake resulted in selection bias. This is done with baseline p-values and also with the Berger-Exner test. See:
- Berger, VW, Exner, DV (1999). “Detecting Selection Bias in Randomized Clinical Trials”, Controlled Clinical Trials 20, 319-327.
- Berger, VW (2005). “Selection Bias and Covariate Imbalances in Randomized Clinical Trials”, John Wiley & Sons, Chichester, Section 6.5.
Thank you for elaborating on this issue and providing additional literature. We agree that, in retrospect, we should have made other choices when designing our (blocked) randomised trial. However, as you also stated, we cannot go back in time. In the revised version of our manuscript, we added p-values to the baseline table (Table 1). Also, we added the limitations of our block randomisation in the Discussion.

Clearly the data are not normally distributed. That could have been determined by appeal to the fact that no data are normally distributed. Why would you feel the need to test to arrive at this conclusion?
>>Our statistical analysis plan was designed to first check if data were normally distributed, so we could know which type of tests to use (parametric or non-parametric). We agree that our data are clearly not normally distributed; however, there is no harm to check this statistically. For clarity for the readers, we removed the first sentence of the section ‘statistical analysis’ for easier reading and more clarity.
There certainly is harm in allowing the folly of normality to hold sway. The data could not have possibly been normally distributed, and pretending that they could be has implications for the preservation of the Type I error rate.
We agree that the data could not have been normally distributed, and thus we changed the text in section *Statistical Analysis*.

Where are the p-values in Table 1?
>>Since patients were randomly distributed across groups, statistical comparison between baseline characteristics is not advocated. In short, if there would be a statistically significant difference, it would be based on chance by definition (since randomised allocation occurred). See for example the Editorial: Statistical testing for baseline differences between randomised groups is not meaningful via https://www.nature.com/articles/s41393-018-0203-y)
This is absolutely incorrect. The randomization is very much in question. Selection bias is very much a possibility. It is this selection bias, enabled by the combination of permuted blocks and unmasking, that we are testing with the baseline p-values. See:
- Berger, VW (2004). “Selection Bias and Baseline Imbalances in Randomized Trials”, Drug Information Journal 38, 1-2.
- Berger, VW (2005). “Selection Bias and Covariate Imbalances in Randomized Clinical Trials”, John Wiley & Sons, Chichester.
- Berger, VW (2009). “Do Not Test for Baseline Imbalances Unless They Are Known To Be Present?”, Quality of Life Research 18, 399.
- Berger, VW (2010). “Testing for Baseline Balance: Can We Finally Get It Right?”, Journal of Clinical Epidemiology 63, 8, 939-940.

Thank you again for your explanation. A stated in our first reply, we added p-values to Table 1.
