# Supplementary material for: Short-term outcomes of cochlear implantation for single-sided deafness compared to bone conduction devices and contralateral routing of sound hearing aids—Results of a Randomised controlled trial (CINGLE-trial)
Source: PLoS One. 2021 Oct 13;16(10):e0257447. doi: 10.1371/journal.pone.0257447 (PMC8513831; doi:10.1371/journal.pone.0257447)
Supplement: Attachment — Submitted filename: Reply to editor_R2.docx [file pone.0257447.s008.docx]

**Reply to editor, revised manuscript PONE-D-20-34585 R2**

To the Academic Editor

Vance Berger, PhD

Dear Academic Editor, dear Vance Berger,

We thank you for your careful consideration of our manuscript. We also thank you for asking a new reviewer for an extra opinion.

In your reply dated July 23^rd^, you asked us to add baseline p-values. However, these values were already added to Table 1 in our most recently uploaded version dated March 12^th^. Based on the comments of the extra reviewer, there were no other points to improve. In the currently uploaded version, I deleted the old reply to reviewers, since no new comments needed to be addressed.

I did include the previous ‘tracked changes’-version of the manuscript, so you can clearly see the addition of the p-values in Table 1.

We hope you consider the current version of our manuscript for publication. Thanks again for your careful consideration.

Please do not hesitate to contact us, should you have any questions or comments.

We hope to hear from you soon. Yours sincerely, on behalf of all authors,

Jeroen P.M. Peters, MD PhD

Corresponding author
